# Rare Delayed Complications of Laparoscopic Adjustable Gastric Band Erosion: Contained Gastric Perforation and Bleeding Pseudoaneurysm

**DOI:** 10.14309/crj.0000000000002309

**Published:** 2026-09-16

**Authors:** Oscar Noble, Ana Paola Islas-Guerra, Jesus Paez-Mayorga, Emiliano Garza-Frias, Davood Abdollahian, Maya Pimentel, Thomas McCarty, Neha Mathur

**Affiliations:** 1 Charles W. Duncan Jr. Department of Medicine, Houston Methodist Hospital, Hoston, TX; 2 Houston Methodist Academic Institute, Houston Methodist Hospital, Hoston, TX; 3 Lynda K. and David M. Underwood Center for Digestive Health, Houston Methodist Hospital, Houston, TX; 4 Department of Interventional Radiology, Houston Methodist Hospital, Houston, TX; 5 Engineering Medicine (EnMed), Texas A&M University, Houston, TX; 6 Weill Cornell Medical College, Cornell University, New York City, NY

**Keywords:** laparoscopic adjustable gastric band, gastric band erosion, melena, endoscopic suturing, pseudoaneurysm

## Abstract

Laparoscopic adjustable gastric banding (LAGB) has declined in use due to long-term complications such as erosions. We report a 65-year-old man who presented with melena 15 years after LAGB placement and ultimately found to have complete intragastric LAGB erosion. His clinical course was complicated by recurrent gastrointestinal bleeding, a contained gastric perforation, intra-abdominal infection, and a right gastric artery pseudoaneurysm. Management required laparoscopic band removal, advanced endoscopic procedures, and arterial embolization. This case highlights a rare but serious late complication of LAGB and emphasizes the need for continued vigilance in patients with legacy bariatric devices.

## INTRODUCTION

Laparoscopic adjustable gastric banding (LAGB) was introduced in the early 1990s as a minimally invasive, reversible procedure for obesity management. By the early 2000s, LAGB was widely adopted due to its perceived safety profile, adjustability, and preservation of gastrointestinal anatomy.^1^ However, long-term outcomes revealed high failure and revision rates, resulting in a substantial decline in the use of LAGB in favor of other bariatric procedures.^2,3^

Common complications of LAGB include band slippage, pouch dilation, esophageal dysmotility, and device malfunction, often requiring reinterventions.^3^ Erosion is a less frequent but clinically significant complication with a prevalence of up to 3.4%.^4–6^ Typical presentations of erosion include abdominal pain, weight regain, and port-site infections. Melena and acute blood loss anemia are rare presenting features, often associated with poor outcomes and complex clinical courses.^7–12^ Endoscopic management of gastric band erosions has had a high percentage of clinical and technical success.^5^

We report a case of gastric band erosion occurring 15 years after LAGB placement, presenting with melena and complicated by contained gastric perforation, recurrent bleeding, and pseudoaneurysm formation.

## CASE REPORT

A 65-year-old man with a history of LAGB placement in 2010 initially presented to an outside emergency department with 4 days of melena and acute blood loss anemia. Abdominal and pelvic computed tomography angiography (CTA) raised concern for band slippage, prompting further evaluation with esophagogastroduodenoscopy (EGD) on day 1, which demonstrated complete intraluminal gastric band erosion with evidence of intraluminal device extension. He received 1 unit of packed red blood cells (pRBC) and was transferred to our facility for a higher-level of care on day 2.

On arrival, the patient was hemodynamically stable, denied abdominal pain, and had a hemoglobin of 8.8 g/dL. He was started on intravenous proton pump inhibitor (PPI) therapy twice daily and was kept nil per os (NPO) pending operative management. On day 3, he underwent laparoscopic removal of the eroded gastric band with lysis of adhesions. Intraoperative findings revealed complete erosion of the band into the stomach, requiring gastrotomy for extraction and primary repair. The procedure was successful, and he was advanced to a clear liquid diet postoperatively.

On day 5, the patient developed a fever to 103.3°F and was started on empiric antibiotics. An upper gastrointestinal series showed no evidence of an active leak. On day 8, he decompensated with hypotension and had a large melenic bowel movement and an episode of hematemesis. His hemoglobin decreased to 7.1 g/dL, prompting the transfusion of 2 units of pRBCs, urgent gastroenterology consultation, and transfer to the medical intensive care unit. Interval CTA showed no active extravasation but suggested retained blood products in the stomach. Antibiotics were broadened, and he remained on intravenous PPI therapy with nasogastric tube (NGT) placement for decompression.

**Figure 1.**
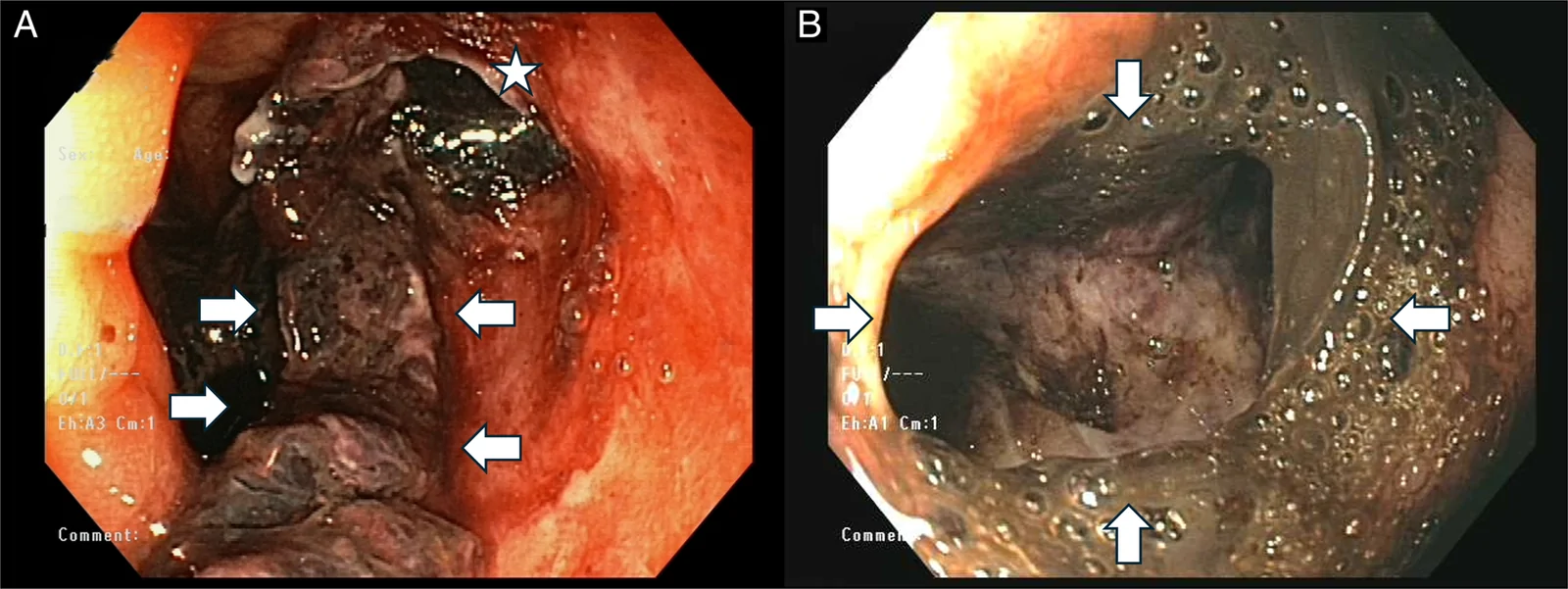
(A) Hospital day 9 EGD with gastric ulceration (star) and large clot burden (arrows). (B) Hospital day 11 EGD with evidence of a better characterized 20 mm gastric wall defect (arrows). EGD, esophagogastroduodenoscopy.

On day 9, repeat EGD revealed a large clot in the proximal stomach, fresh blood, and a 10 mm nonbleeding gastric wall defect at the surgical site with diffusely friable mucosa with contact bleeding (Figure 1). Hemostatic spray was applied. He remained NPO with NGT, with adjunctive metoclopramide and sucralfate therapy. An interval EGD performed on day 11 revealed a reduced clot burden and a more discernible 20 mm gastric wall defect (Figure 1). Follow-up abdominal and pelvic computed tomography with oral and intravenous contrast demonstrated a full-thickness 1.3 × 1.2 cm defect with extraluminal gas but no contrast extravasation, consistent with a contained perforation. No immediate surgical intervention was pursued.

**Figure 2.**
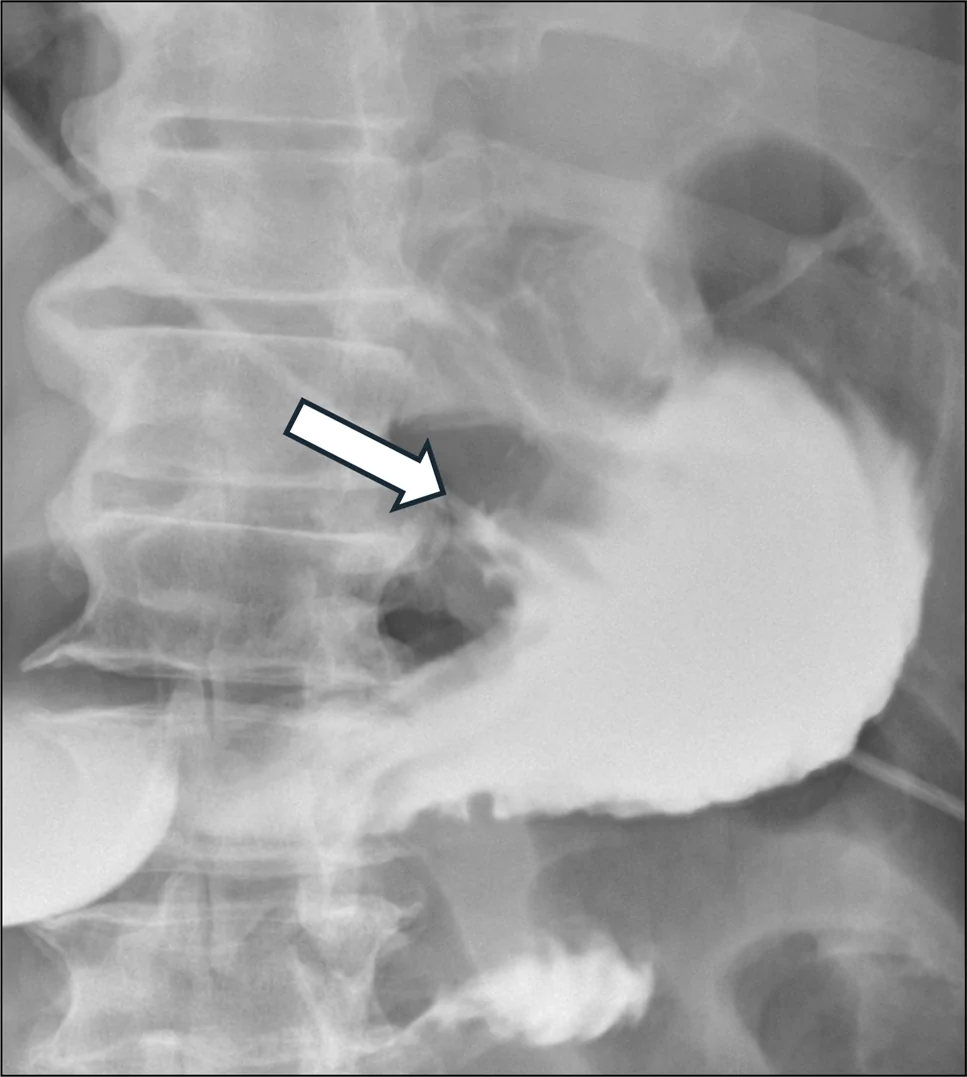
Fluoroscopy upper gastrointestinal series with residual contrast-filled outpouching (arrow) from the lesser curvature of the stomach consistent with contained perforation.

The patient remained clinically stable without peritoneal signs and was managed conservatively with NPO, NGT, and PPI therapy. Total parenteral nutrition was initiated on day 12. Repeat upper gastrointestinal series on day 13 showed a small, contrast-filled outpouching along the lesser curvature of the stomach without extravasation (Figure 2). On day 17, he experienced another melenic bowel movement with a decline in hemoglobin, prompting evaluation by an interventional gastroenterologist.

**Figure 3.**
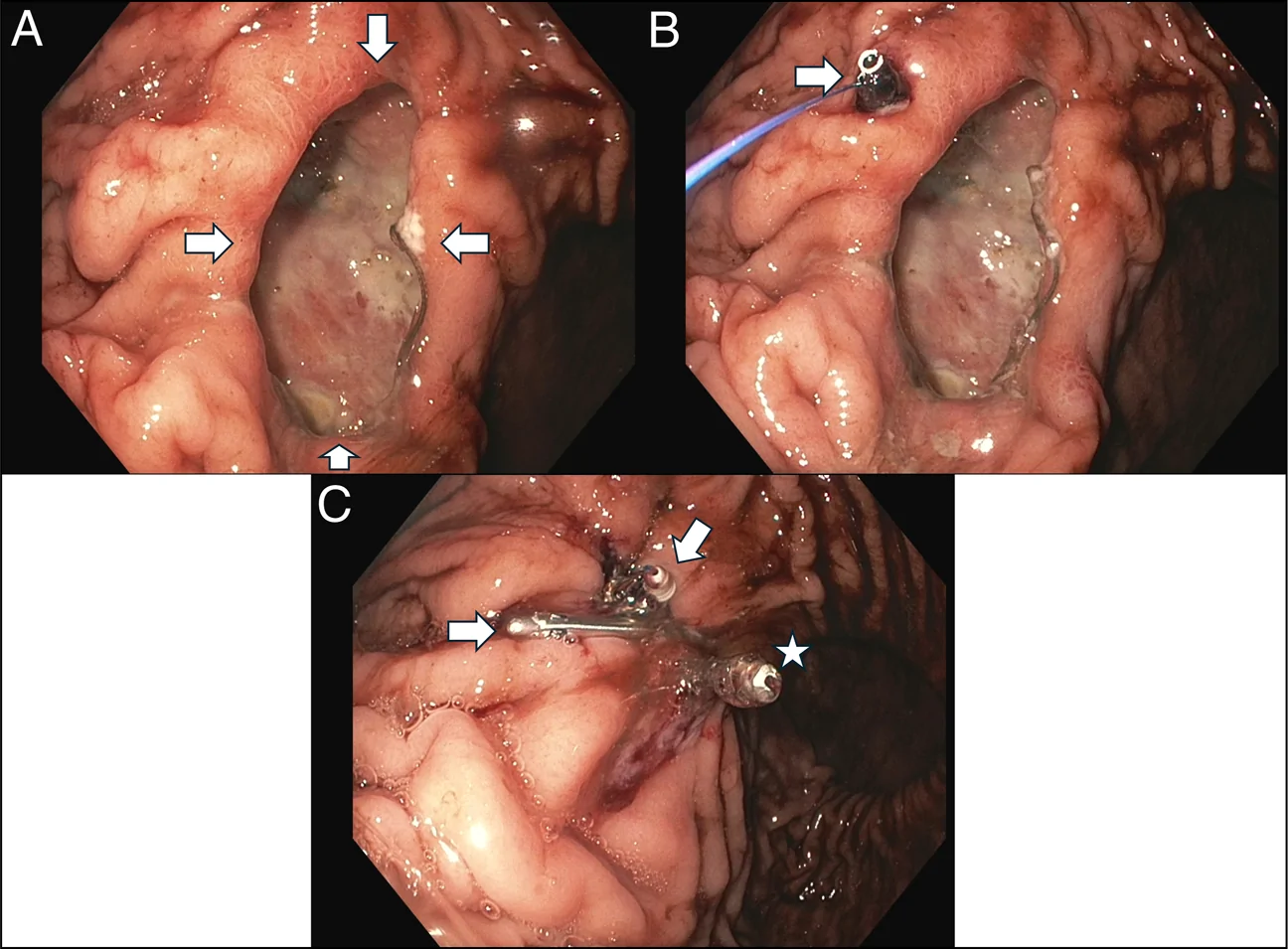
Hospital day 18 esophagogastroduodenoscopy. (A) Severe 50 mm cratered gastric ulceration (arrows). (B) Helical tack (arrow) placement around the gastric ulceration. (C) Repaired ulceration after 4 helical tacks (arrows) and a hemostatic clip (star) were placed.

**Figure 4.**
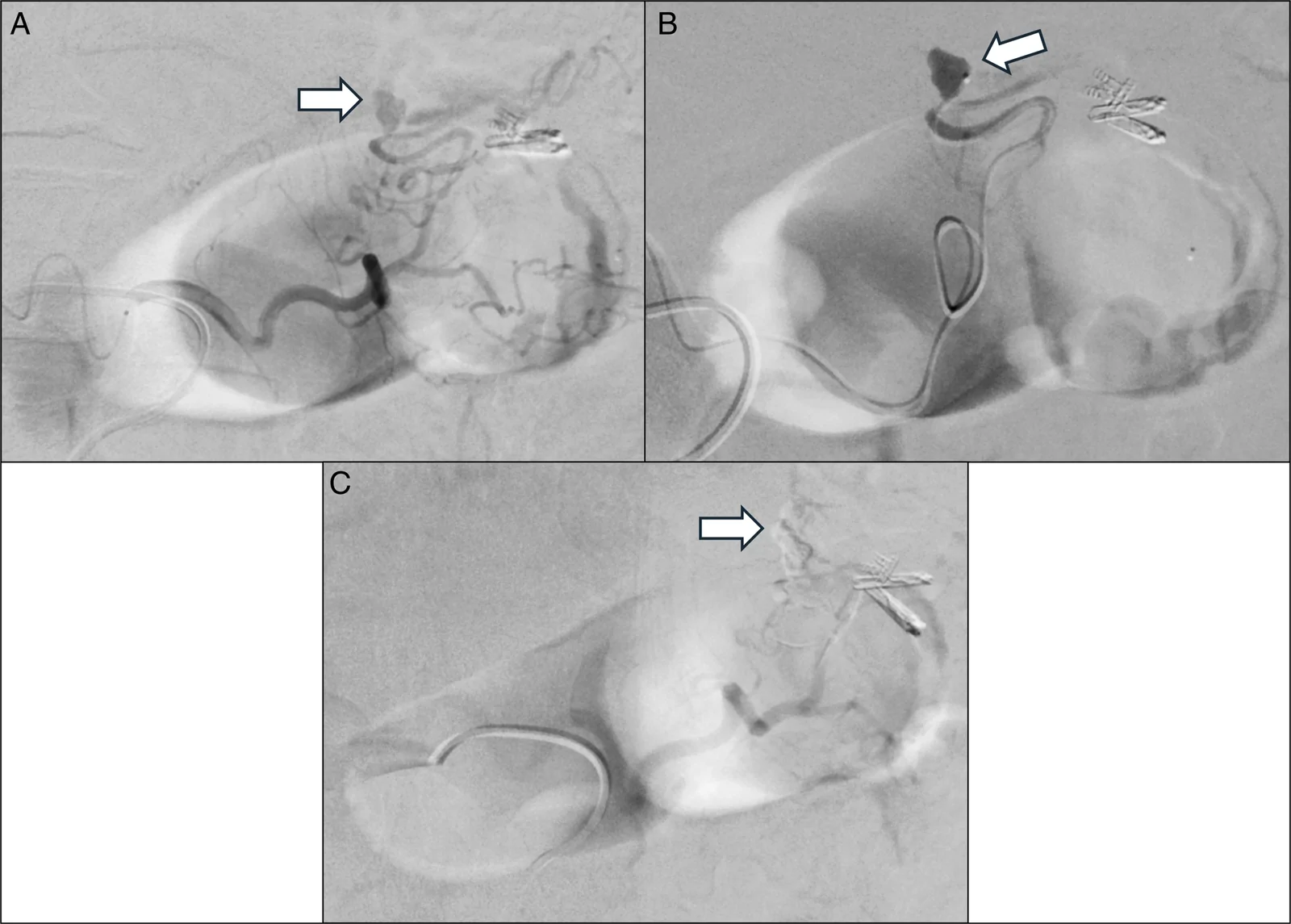
(A) Arteriogram of the proximal right gastric artery and its distal branches. A pseudoaneurysm (arrow) measuring approximately 0.7 cm × 0.5 cm was seen within a superior distal branch. (B) A microcatheter was advanced into the pseudoaneurysm (arrow) and an arteriogram was performed to confirm its location before N-butyl cyanoacrylate glue embolization. (C) Postembolization arteriogram from the base catheter within the common hepatic artery with complete occlusion of the distal right gastric artery pseudoaneurysm.

On day 18, a third EGD revealed a 50 mm cratered gastric ulcer with dehiscence at the erosion site. Hemostatic gel was injected, and endoscopic suturing was performed using a helical tack-based system with clip reinforcement and endoscopic sealant application without procedural complications (Figure 3). On day 22, his hemoglobin decreased to 6.9 g/dL, requiring transfusion of 2 pRBC units. Interventional radiology performed celiac and hepatic arteriography, identifying an occluded left gastric artery, likely secondary to the LAGB, and a hypertrophied right gastric artery with a distal pseudoaneurysm (Figure 4)**,** which was successfully managed with glue embolization.

Repeat CTA on the same day showed no active extravasation but revealed extensive pulmonary emboli. Intravenous heparin was started, and the patient was transferred to the medical intensive care unit for hemodynamic monitoring. On day 26, the patient tolerated diet, total parenteral nutrition was discontinued, his hemoglobin remained stable, heparin was transitioned to apixaban, and he was transferred to the floor. He was discharged on hospital day 27. The last outpatient follow-up was documented on postdischarge day 116 with no further episodes of melena, hematemesis, or fever, with an improving hemoglobin level and healing postoperative abdominal wounds (Table 1).

**Table 1.** Clinical timeline from prehospitalization to last postdischarge follow-up

Day	Key event
Pre-hospitalization	
Days 1–4	Painless melena
Hospital	
Day 1	EGD: gastric band erosion with intraluminal extension; 1u pRBC transfused
Day 2	Transferred to higher level care facility; IV PPI, NPO
Day 3	Laparoscopic gastric band removal with gastrotomy and primary repair
Day 5	Fever; antibiotics started; upper GI series negative for leak
Day 8	GI bleed (melena) with hypotension; 2u pRBC; transferred to ICU
Day 9	EGD: large gastric clot, friable mucosa, 10 mm wall defect; hemostatic spray used
Day 11	CT: contained perforation, no extravasation
Day 12	TPN started
Day 17	Large melenic stool; acute hemoglobin drop
Day 18	EGD: 50 mm cratered ulcer with dehiscence; endoscopic suturing, clipping, and sealant
Day 22	Drop in hemoglobin. CT angiography: right gastric artery pseudoaneurysm s/p glue embolization. Postprocedure CTA without bleed but incidental pulmonary emboli. IV heparin started; ICU transfer for monitoring
Day 26	Hemoglobin stable; transitioned to apixaban; tolerating diet; TPN stopped; transferred to floor
Day 27	Discharged with stable hemoglobin, tolerating diet, no melena
Post-discharge	
Day 35	ID follow-up; prolonged antibiotics for postoperative wounds
Day 96	Wound debridement and drainage
Day 116	ID follow-up; hemoglobin improving; cultures negative; wounds healing; completing antibiotic course

CT, computed tomography; CTA, computed tomography angiography; EGD, esophagogastroduodenoscopy; GI, gastrointestinal; ICU, intensive care unit; ID, infectious disease; IV, intravenous; NPO, nothing by mouth; PPI, proton-pump inhibitor; pRBC, packed red blood cells; TPN, total parenteral nutrition.

## DISCUSSION

This case highlights delayed erosion occurring 15 years after LAGB placement and the uncommon presentation and complications that produced a prolonged, complex clinical course. Although most erosions occur within the first few years after surgery, delayed presentations beyond a decade have been reported.^3,6,8,11^ Although previous reports have described gastrointestinal bleeding and pseudoaneurysm formation in association with LAGB erosion,^9^ the distinguishing feature of this case is the presence of recurrent bleeding despite band removal, failed conservative management, and advanced endoscopic repair of the gastric wall defect, with definitive hemostasis achieved only after angiographic identification and embolization of an initially unidentified distal right gastric artery pseudoaneurysm. This sequence suggests that vascular injury may remain clinically relevant even after the inciting foreign body has been removed, and that persistent bleeding after apparent local source control should prompt evaluation for a vascular complication.

Mechanistically, visceral artery pseudoaneurysms are known to arise from arterial wall disruption in the setting of trauma, surgery, infection, or inflammation, which can be iatrogenic in origin and with a known mean time of presentation of 6 weeks after insult.^13,14^ In this patient, the angiographic findings of an occluded left gastric artery and hypertrophied right gastric artery with distal pseudoaneurysm suggest chronic vascular compromise with collateralization likely resulting from gradual band erosion into the vasculature, which contributed to delayed healing and recurrent bleeding despite multiple failed previous interventions.

This case underscores the importance of maintaining a high index of suspicion for late LAGB-related complications in patients presenting with gastrointestinal bleeding, even in the absence of abdominal symptoms. Diagnosis typically relies on cross-sectional imaging and endoscopy, and management may necessitate a multidisciplinary approach involving surgical, endoscopic, and interventional radiology teams, as in our case. Importantly, in patients with recurrent bleeding after band removal or endoscopic closure, clinicians should have a low threshold to consider early angiographic evaluation to assess for vascular complications, particularly in the setting of chronic erosion, perforation, or unexplained recurrent hemorrhage. In our case, the culprit was not identified until after multiple failed interventions and repeat episodes of bleeding.

As the population with legacy bariatric devices ages, awareness of rare but serious delayed complications becomes essential. This case adds to the limited literature describing melena, perforation, and vascular complications as manifestations of LAGB erosion and reinforces the need for long-term surveillance.

## DISCLOSURES

Author contributions: All authors contributed to the writing and review of this case report. The final draft was read and approved by all authors. N. Mathur is the article guarantor.

Financial disclosure: None to report.

Informed consent was obtained for this case report.

## ABBREVIATIONS:

CTA: computed tomography angiography
EGD: esophagogastroduodenoscopy
LAGB: laparoscopic adjustable gastric banding
NGT: nasogastric tube
NPO: nil per os
PPI: proton pump inhibitor
pRBC: packed red blood cells
